# The Role of Muscle Perfusion in the Age-Associated Decline of Mitochondrial Function in Healthy Individuals

**DOI:** 10.3389/fphys.2019.00427

**Published:** 2019-04-12

**Authors:** Fatemeh Adelnia, Donnie Cameron, Christopher M. Bergeron, Kenneth W. Fishbein, Richard G. Spencer, David A. Reiter, Luigi Ferrucci

**Affiliations:** ^1^Translational Gerontology Branch, Intramural Research Program, National Institute on Aging, National Institutes of Health, Baltimore, MD, United States; ^2^Norwich Medical School, University of East Anglia, Norwich Research Park, Norwich, United Kingdom; ^3^Laboratory of Clinical Investigation, Intramural Research Program, National Institute on Aging, National Institutes of Health, Baltimore, MD, United States; ^4^Department of Radiology and Imaging Sciences, Emory University, Atlanta, GA, United States

**Keywords:** bioenergetic, muscle perfusion, peak-VO_2_, aging, ^31^P MRS, diffusion weighted MRI

## Abstract

Maximum oxidative capacity of skeletal muscle measured by *in vivo* phosphorus magnetic resonance spectroscopy (^31^P-MRS) declines with age, and negatively affects whole-body aerobic capacity. However, it remains unclear whether the loss of oxidative capacity is caused by reduced volume and function of mitochondria or limited substrate availability secondary to impaired muscle perfusion. Therefore, we sought to elucidate the role of muscle perfusion on the age-related decline of muscle oxidative capacity and ultimately whole-body aerobic capacity. Muscle oxidative capacity was assessed by ^31^P-MRS post-exercise phosphocreatine recovery time (τ_PCr_), with higher τ_PCr_ reflecting lower oxidative capacity, in 75 healthy participants (48 men, 22–89 years) of the Genetic and Epigenetic Signatures of Translational Aging Laboratory Testing study. Muscle perfusion was characterized as an index of blood volume at rest using a customized diffusion-weighted MRI technique and analysis method developed in our laboratory. Aerobic capacity (peak-VO_2_) was also measured during a graded treadmill exercise test in the same visit. Muscle oxidative capacity, peak-VO_2_, and resting muscle perfusion were significantly lower at older ages independent of sex, race, and body mass index (BMI). τ_PCr_ was significantly associated with resting muscle perfusion independent of age, sex, race, and BMI (*p*-value = 0.004, β = −0.34). τ_PCr_ was also a significant independent predictor of peak-VO_2_ and, in a mediation analysis, significantly attenuated the association between muscle perfusion and peak-VO_2_ (34% reduction for β in perfusion). These findings suggest that the age-associated decline in muscle oxidative capacity is partly due to impaired muscle perfusion and not mitochondrial dysfunction alone. Furthermore, our findings show that part of the decline in whole-body aerobic capacity observed with aging is also due to reduced microvascular blood volume at rest, representing a basal capacity of the microvascular system, which is mediated by muscle oxidative capacity. This finding suggests potential benefit of interventions that target an overall increase in muscle perfusion for the restoration of energetic capacity and mitochondrial function with aging.

## Introduction

The progressive decline in mobility negatively affects the quality of life in older persons and may ultimately lead to disability and frailty (Ferrucci et al., 2016; Hall et al., 2017). While the etiology of impaired mobility is multifactorial, loss of muscle strength and aerobic capacity are important contributors (Kalyani et al., 2014; Ferrucci et al., 2016). Recently, we demonstrated that the age-related decline of whole-body aerobic capacity (peak VO_2_ uptake) is accounted for, at least in part, by the decline of the *in vivo* maximum oxidative capacity of skeletal muscle with age, assessed by ^31^P-MRS post-exercise phosphocreatine (PCr) recovery time (τ_PCr_) (Choi et al., 2016), which largely reflects maximal capacity of mitochondria to synthesize adenosine triphosphate (ATP) (Arnold et al., 1984; Prompers et al., 2014). A caveat to this marker is that the rate of PCr resynthesis reflects the intrinsic oxidative capacity of mitochondria only under the assumption that sufficient changes in muscle perfusion allow the delivery of adequate amount of oxygen and substrate to mitochondria (Kemp et al., 2002; Kent and Fitzgerald, 2016). Although there is compelling evidence from respirometry conducted in permeabilized human muscle fibers that mitochondrial function intrinsically declines with age (Short et al., 2005; Mellem et al., 2017), we cannot exclude the possibility that changes in muscle perfusion also play an important role in the age-associated decline of mitochondrial function and ultimately whole-body aerobic capacity. For example, it has been suggested that age (Lawrenson et al., 2004; Hearon and Dinenno, 2016; Hildebrandt et al., 2017) and disease (Gueugneau et al., 2016; Wagenmakers et al., 2016; Creager, 2018) may impair the transport of oxygen and nutrients because of a reduction in cardiac function or impairment of peripheral vascular adaptation. Indeed, a wealth of data suggest that both capillary density (Urbieta-Caceres et al., 2012; Barnouin et al., 2017) and vasodilation that occurs under metabolic demand substantially decrease in older adults (Mueller et al., 2017; Tonson et al., 2017). However, few studies have directly examined the relative contribution of basal or dynamic changes in muscle perfusion to oxidative capacity of skeletal muscle and whole-body aerobic capacity.

The study of muscle perfusion *in vivo* has been addressed with different methods that are technically challenging, particularly at rest, and none of which is fully satisfactory. For example, near infrared spectroscopy (NIRS) can be used to measure blood flow in a local site, however, it is sensitive to the interface of subcutaneous fat or non-metabolically active tissues, and thus requires careful calibration, particularly when used in longitudinal studies of older adults (Jones et al., 2016). Other methods based on pressure transducers also lack sensitivity and capture only part of capillary flow (Dinenno et al., 2001).

In this work we employed a magnetic resonance imaging (MRI) method based on diffusion weighted imaging (DWI) (Le Bihan et al., 1986; Cameron et al., 2017) that estimates an index of microvascular blood volume at the capillary level and uses it as a marker of muscle perfusion at rest.

The primary objective of this cross-sectional study was to investigate the role of resting muscle perfusion in both age-associated decline of mitochondrial oxidative capacity and whole-body aerobic capacity in a population of healthy individuals. A second goal was to examine whether the association between muscle perfusion and aerobic capacity is mediated by mitochondrial oxidative capacity. Thus, we tested three specific hypotheses: (i) resting muscle perfusion is correlated to the muscle oxidative capacity independent of the potential confounding effects of age, sex, race, and body mass index (BMI); and (ii) resting muscle perfusion is also correlated with whole-body aerobic capacity (peak VO_2_) independent of the potential confounding effects of age, sex, race, and BMI; and (iii) the association between muscle perfusion and peak VO_2_ is mediated by mitochondrial function.

## Materials and Methods

### Participants

Participants were selected from the Genetic and Epigenetic Signatures of Translational Aging Laboratory Testing (GESTALT) study, which is sponsored and conducted by the Intramural Research Program (IRP) of the National Institute on Aging (NIA) at the clinical research center located at Harbor Hospital (Baltimore, MD, United States) under the approval and oversight of the IRB of the National Institute of Environmental Health Science (Research Triangle Park, NC, United States). Briefly, GESTALT was designed to evaluate and cross-correlate biological, phenotypical, and functional differences that occur with age in healthy individuals across a wide age-range. Participants were screened for eligibility by trained medical personnel and were enrolled in the study based on the following inclusion criteria: no established genetic disease; no autoimmune disease; no history of cardiovascular, kidney, liver, or neurological diseases, or diabetes; no active cancer; no significant hormonal dysfunction; no chronic muscle pain; no chronic drug treatment except one antihypertensive drug (with controlled blood pressure); capacity to perform normal activities of daily living without shortness of breath; and ability to walk independently for at least 400 meters. We studied 75 healthy adults from GESTALT, with sample characteristics shown in Table 1. Participants underwent a 3-day comprehensive examination (between 2015 and 2018) including physical examination, health history assessment, ^31^P MRS and diffusion weighted MRI during their first GESTALT visit in the Clinical Research Unit of the National Institute on Aging Intramural Research Program. Trained technicians performed all measurements following standardized protocols at the baseline visit of the GESTALT study.

**Table 1:** Descriptive characteristics of the study sample.

Characteristic	Mean *(SD)*
Number of participants	75
Age, [years, range]	52.06 [22-89]
Sex, Male-to-female ratio (%)	62.34
Race, African-American (%)	13.3
Height, cm	172.53 (9.39)
Weight, kg	76.70 (11.75)
Body mass index, kg/m^2^	25.67 (2.58)
Systolic blood pressure, mm Hg	118.27 (12.39)
Diastolic blood pressure, mm Hg	74.73 (7.75)
Peak VO_2_, mL/kg/min	28.65 (6.30)
τ_PCr_ (sec)	45.85 (11.93)
Muscle perfusion (index of blood volume)	0.54 (0.015)

### Aerobic Capacity (Peak VO_2_)

The aerobic capacity was measured with a modified version of the treadmill Balke test (Fleg et al., 2005) as previously described. Briefly, participants walked at a constant speed throughout the test when the slope of treadmill was initially set to 0%, and increased by 3% every 2 min until voluntary exhaustion, leg pain or the development of these symptoms. Oxygen consumption was assessed every 30 s and the highest value was considered as peak VO_2_.

### Diffusion-Weighted MRI and ^31^P MRS

Diffusion–weighted (DW) imaging and ^31^P spectroscopy data were collected in participants using a 3T Philips Achieva MR scanner (Philips, Best, Netherlands) in the same imaging session. For DW data acquisition, participants were positioned supine, feet-first with a 10 cm bolster pillow under the knees to align legs parallel to the scanner’s bore. Data were collected with a 32-channel cardiac coil using a single 22 mm axial slice placed right above the middle of the left thigh after participants had been lying down resting for 30 min. A diffusion weighted spin echo sequence with a single-shot echo planar imaging readout was applied with the following parameters: TR/TE = 3000/61.48 ms; FOV = 225.28 mm × 256 mm; matrix size = 224 × 224; parallel imaging acceleration factor 2; partial Fourier factor 0.6 in the phase-encoding direction; 8 signal averages; *b*-values = 0, 50, 300, 475, 754 s/mm^2^; with diffusion gradients along the slice, phase and read directions and a triple fat suppression technique developed in our laboratory (Cameron et al., 2017). The scan time for each direction was 3 min and 25 s, resulting in a total scan time of 10 min and 15 s.

To characterize an *in vivo* index of muscle perfusion at rest, we assumed that the superdiffusive transport of blood through tissue occurs according to the continuous time random walk (CTRW) model (Ingo et al., 2014, 2015). While the physical basis for the application of the CTRW to the problem of transport within the vascular compartment has not been completely formalized, it provides strong motivation to view this system as exhibiting anomalous diffusion. We therefore employed the stretched exponential (SE) form, and interpret the stretching exponent fit based on the smaller *b-*values (ranging from 0 to 800 s/mm^2^) of a DWI signal (Seo et al., 2018; Tang and Zhou, 2019) inversely reflects the microvascular blood volume (Lenz et al., 2010; Reiter et al., 2018). The DWI signals were therefore fit to the SE function:

(1) $$\text{S}(\text{b})/{\text{S}}_{0}=\exp[-{\left(\text{b}{\text{D}}^{\prime }\right)}^{\text{β}/2}]$$

where *S* is the signal at different diffusion-weighting values (*b*), *S*_0_ is the signal without diffusion weighting, *D*′ is the effective diffusion coefficient of water molecule in tissue, and β is the stretching parameter, which can range from 0 to 2. In fact, the stretching exponent in both CTRW and SE models characterize the water molecular motions that deviate from normal Brownian motion (Hall and Barrick, 2008; Ingo et al., 2014). In this formalism, β = 2 reflects normal Brownian motion and deviations of β to values less than 2 reflect superdiffusion (Ingo et al., 2014). Therefore, smaller values of β represent a greater deviation from Brownian motion. For simplicity, we will use 1/β as an index for microvascular blood volume which represent the muscle perfusion. With the focus of this work on muscle perfusion, we will limit our attention to the stretching exponent of Equation (1) as it relates to energetic capacity of muscle. To calculate 1/β, region of interest (ROI) analysis was performed using in-house MATLAB (MathWorks, Natick, MA, United States) scripts. ROIs were manually drawn in the region of the vastus lateralis muscle that coincided with the ^31^P MRS measurement for each participant, and were carefully selected to exclude large vessels, fascia, and fat. The average ROI signal intensity for each orthogonal diffusion direction was normalized to the *S*_0_ of that ROI and then was fit with Equation (1). The mean β for each individual was calculated by averaging β values from all three orthogonal diffusion sensitization directions.

**FIGURE 1:**
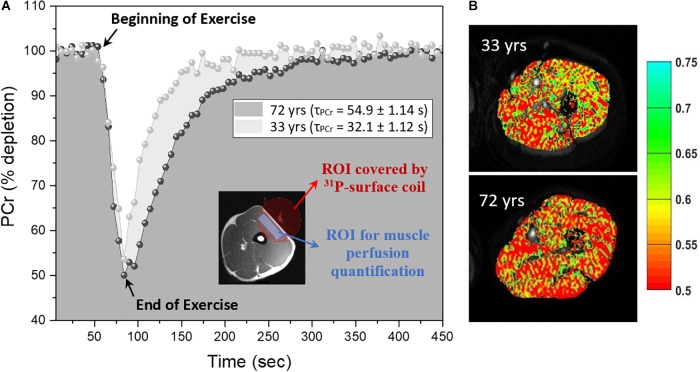
Representative phosphocreatine (PCr) curves and index of blood volume maps **(A)** Right side: PCr curves before, during and after exercise for a young (33 year-old) and old (72 year-old) male, the inset shows an example of thigh muscle with the region of interest (ROI) covered by 10-cm ^31^P tuned surface coil in red and approximate region used in ROI drawing for muscle perfusion quantification at rest in blue. **(B)** Left side: Corresponding diffusion-weighted imaging that have been superimposed with the perfusion map measured as the index of blood volume (1/β) calculated based on equation (1) as discussed in the text for the slice diffusion sensitization.

^31^P-MRS data were acquired after DW imaging data acquisition using a 10-cm ^31^P-tuned surface coil (PulseTeq, Surrey, United Kingdom) that was fastened above the middle of the left thigh covering the DWI slice position over the vastus lateralis muscle. The spatial localization of the ^31^P MRS signal was limited to the sensitive region of the coil as shown in the inset of Figure 1A which primarily consisted of the vastus lateralis muscle with minimal contribution of signal from adjacent muscles.

Briefly, participants performed a rapid ballistic knee extension exercise for approximately 25–50 s while a total of 75 pulse-acquire ^31^P-MRS spectra were collected before, during, and after exercise, resulted in a total 450 s scan time, as shown in Figure 1A (Choi et al., 2016). Acquisition parameters included sampling frequency of 2048 HZ, a 1.5 s repetition time and four signal averages, totaling a 6 s time-resolution. Exercise duration was defined by two criteria: (i) achieving a reduction in PCr peak height of 30–80% compared with initial baseline values and (ii) avoidance of intracellular muscle acidification, defined as pH < 6.8 (Paganini et al., 1997).

The maximum oxidative capacity was assessed during the recovery period immediately following exercise, with the rate of PCr recovery post-exercise reflecting maximal *in vivo* muscle oxidative ATP synthesis (Prompers et al., 2014). As previously described (Prompers et al., 2014; Choi et al., 2016) a mono-exponential function was used to fit the time-dependent post-exercise PCr recovery:

(2) $$\text{PCr}(\text{t})={\text{PCr}}_{0}+\Delta \text{PCr}[1-\exp(-\text{t}/{\text{τ}}_{\text{PCr}})]$$

where PCr_0_ is the amplitude of PCr at the commencement of recovery, ΔPCr is the decrease of PCr during exercise, and τ_PCr_ is the recovery time constant of PCr. The τ_PCr_ parameter is inversely proportional to the maximum *in vivo* oxidative capacity of skeletal muscle; namely, oxidative ATP synthesis, with a negligible contribution from anaerobic metabolism (Arnold et al., 1984; Edwards et al., 2012).

### Statistical Analysis

Simple linear regression models were used to explore relationships of τ_PCr_, resting muscle perfusion, and peak VO_2_ with age. Multivariate regression models were used to explore the relationship between τ_PCr_ as the dependent variable and muscle perfusion as independent variable after adjusting for age, sex, race, and BMI. The correlations of peak VO_2_ with perfusion and τ_PCr_ were separately and then jointly assessed using linear regression analyses. The interaction between τ_PCr_ and muscle perfusion was tested to understand whether the effect of τ_PCr_ on peak VO_2_ is different at different levels of perfusion. The Sobel test (Imai et al., 2014) was performed to evaluate whether muscle oxidative capacity (τ_PCr_) mediates the effect of resting muscle perfusion on aerobic capacity. All analyses were performed using R (Version 3, R Foundation for Statistical Computing, Vienna, Austria). Standardized regression coefficients were obtained from regression models, and *p* < 0.05 was considered statistically significant in all analyses.

## Results

The cross-sectional study sample included 75 adults with a mean age of 52.06 years range from 22 to 89 years old. The main characteristics of the participants enrolled in this study are reported in Table 1 and reflect the exceptional health status of this cohort. Representative post-exercise ^31^P MRS PCr recovery curves in Figure 1A show a shorter recovery time for a young (33 year-old) male compared with an older (72 year-old) male. Figure 1B shows corresponding maps of the DW imaging index of blood volume based on Equation (1).

The recovery time of PCr peak following exercise (τ_PCr_) was significantly greater (standardized β = 0.338, *p*-value = 0.003) in older adults as shown in Supplementary Figure 1a, consistent with the previously described decrease in muscle oxidative capacity with older age, which is confirmed even in this extremely healthy population. This association remained significant even after adjustment for sex, race and BMI (Model A.1, Table 2). Resting muscle perfusion was significantly lower with older age independent of BMI, sex, and race (standardized β = −0.329, 95% CI = −0.549, −0.108, *p*-value = 0.004). As expected, peak VO_2_ was also progressively lower with older age, shown in Supplementary Figure 1c, independent of BMI, sex, and race (standardized β = −0.619, 95% CI = −0.802, −0.436, *p*-value < 0.001).

**Table 2:** Linear regression models testing association of τ_PCr_ with age (model A.1), with muscle perfusion (model A.2) and both age and muscle perfusion (model A.3).

	Model A.1; τ_PCr_ = Age		Model A.2; τ_PCr_ = Perfusion		Model A.3; τ_PCr_ = Age + Perfusion	

adj. *R*^2^	0.073		0.125		0.163	

parameters	B (95% CI)	*P*-value	B (95% CI)	*P*-value	B (95% CI)	*P*-value
Age (years)	0.354 (0.127, 0.581)	0.003^∗^			0.237 (0.007, 0.467)	0.044^∗^
Perfusion (index of blood volume)			−0.414 (−0.631, −0.196)	<0.001^∗^	−0.332 (−0.56, −0.105)	0.005^∗^

*All models are additionally adjusted for BMI, sex, and race (not shown here). Coefficients are standardized. ^∗^Statistically significant.*

**FIGURE 2:**
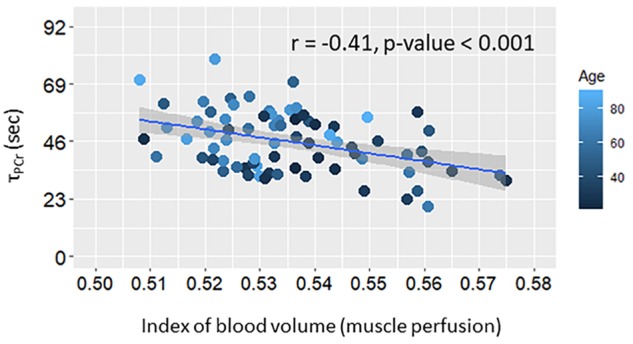
Scatter plot of post-exercise recovery time of phosphocreatine (τ_PCr_) versus muscle perfusion at rest. Linear regression line and summary statistic (Pearson’s correlation and *p*-value) are shown. Color gradient specifies the age of each single plotted participant.

The index of blood volume at rest was significantly correlated with the reduced muscle oxidative capacity over all subjects (Model A.2, Table 2) as shown in Figure 2, and the association remained statistically significant after adjusting this analysis for age (Model A.3; Table 2). There was no significant interaction between age and perfusion in Model A.3 (analyses not shown) suggesting that the independent effect of resting muscle perfusion on muscle oxidative capacity is similar across the entire age range in this sample of very healthy individuals.

**FIGURE 3:**
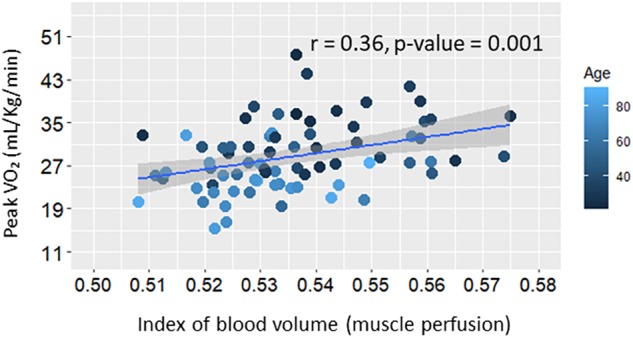
Scatter plot of whole-body aerobic capacity (Peak VO_2_) versus muscle perfusion at rest. Linear regression line and summary statistic (Pearson’s correlation and *p*-value) are shown. Color gradient specifies the age of each single plotted participant.

**FIGURE 4:**
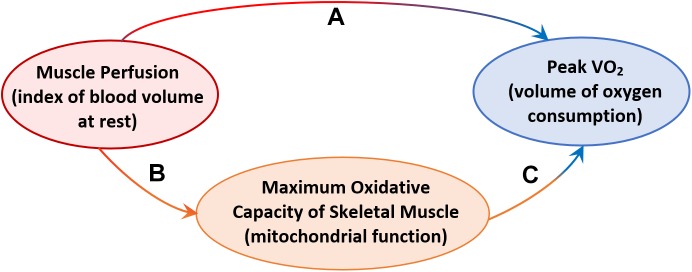
Diagram illustrating: **(A)** the demonstrated effect of resting muscle perfusion on whole-body aerobic capacity (Peak VO_2_), **(B)** the effect of resting muscle perfusion on muscle oxidative capacity measured as post-exercise recovery time of phosphocreatine (τ_PCr_), **(C)** the effect of resting muscle perfusion on Peak VO_2_, mediated by τ_PCr_.

Further analyses were aimed at testing the hypothesis that resting skeletal muscle perfusion correlates with peak VO_2_ and that this association is mediated, at least in part, by mitochondrial function. Peak VO_2_ was significantly higher in adults with higher muscle perfusion, as shown in Figure 3, and the association was still statistically significant after adjusting for covariates (Model B.1, Table 3). In agreement with previous findings (Choi et al., 2016), we found that peak VO_2_ is correlated with maximum oxidative capacity of skeletal muscle in this healthy population even after accounting for covariates (Model B.2, Table 3). In linear regression models that included both τ_PCr_ and perfusion (Model B.3, Table 3), the relationship between muscle perfusion and peak VO_2_ was attenuated by the inclusion of τ_PCr_ (with an approximate reduction of 34%). In contrast, maximum oxidative capacity remained significant with an estimated reduction of about 16%. This mediation of mitochondrial oxidative capacity was confirmed by significant (*p*-value < 0.02) Sobel test statistic with an indirect effect of 29.79 (95% CI = 5.44, 71.29), as illustrated in Figure 4. It is worth noting that no significant interaction was observed between τ_PCr_ and muscle perfusion in Model B.3 (analyses not shown).

**Table 3:** Linear regression models testing association of Peak VO_2_ with muscle perfusion (model B.1), with τ_PCr_ (model B.2) and both muscle perfusion and τ_PCr_ (model B.3).

	Model B.1; Peak VO_2_ = perfusion		Model B.2; Peak VO_2_ = τ_PCr_		Model B.3; Peak VO_2_ = τ_PCr_ + perfusion	

adj. *R*^2^	0.523		0.541		0.548	

parameters	B (95% CI)	*P*-value	B (95% CI)	*P*-value	B (95% CI)	*P*-value
Age (years)	−0.575 (−0.75, −0.402)	<0.001^∗^	−0.564 (−0.738, −0.390)	<0.001^∗^	−0.535 (−0.713, −0.358)	<0.001^∗^
τ_PCr_ (sec)			−0.256 (−0.429, −0.0837)	0.004^∗^	−0.216 (−0.396, −0.035)	0.019^∗^
Perfusion (index of blood volume)	0.193 (0.022, 0.365)	0.027^∗^			0.128 (−0.05, 0.305)	0.156

*All models are adjusted for BMI, sex, and race (not shown here). Models B.2 and B.3 are additionally adjusted for PCr depletion (not shown here). Coefficients are standardized. ^∗^Statistically significant.*

## Discussion

In this study we show that τ_PCr_ increases with age, indicating a decline in skeletal muscle oxidative capacity, as measured by ^31^P MRS. This finding is consistent with previous work (Choi et al., 2016; Zane et al., 2017) and, given the exceptional health status of GESTALT participants, strongly supports the notion that aging is associated with decline in *in vivo* oxidative capacity of skeletal muscle, even when a population of individuals who are free of clinically detectable diseases is selected. Using DWI, we also show that resting muscle perfusion assessed by the index of blood volume as a proxy measure, declines with aging. The interpretation of the effect of our measured perfusion parameter is relatively straightforward, as it can be directly connected with a decline in capillary density and an increase in microvascular resistance or stiffness that leads to an overall reduction in blood volume in skeletal muscle at rest. This finding is consistent with the work of Lawrenson et al. (2003), which showed that microvascular resistance in the leg increases with advancing age. Authors of that work argued that the elevated vascular resistance in the leg in older adults, as measured using a pressure transducer during knee extensor exercise, is likely responsible for quadriceps blood flow attenuation in older subjects and that this may be the result of microvasculature impairment at rest. In addition, Tonson et al. (2017) showed that the peak blood-oxygen-level-dependent (BOLD) microvasculature responses were significantly decreased in older adults compared with younger adults in calf muscle after 1-s maximal voluntary isometric contraction, likely due to microvasculature dysfunction. In fact, a global consensus has not been yet reached to explain the mechanisms by which this age-associated decline of peripheral vascular function occurs with age. For example, it has been suggested that age-associated reduction in muscle perfusion may be explained in part by changes in arterial function (Behnke and Delp, 2010; Ward et al., 2018), endothelial dysfunction and/or impairment of the endothelial NAD^+^-H_2_S signaling network with aging (Das et al., 2018) and a decline in the number of capillaries in aging muscle (Urbieta-Caceres et al., 2012; Bigler et al., 2016). Nevertheless, our finding remains important because it directly measures an index of resting *in vivo* microvascular blood volume, suggesting a direct impact of reduced number of capillaries and/or increase of microvasculature resistance with aging.

The key finding of this work is the empirical demonstration that part of the decline in maximum oxidative capacity of muscle with healthy aging stems from a reduction in blood volume that is already detectable at rest, and this correlation remains significant after adjusting for age and other cofounders. In fact, resting blood volume can be postulated as a basal capacity (e.g., capillary density and microvascular resistance) of vasodilation in response to the cellular metabolic demand (Lawrenson et al., 2003; McGuire and Secomb, 2003), whereby having an adequate basal capacity augments sufficient vasodilation. These results thus demonstrate that the age-associated decline of resting muscle perfusion may act, at least in part, as a causative factor in the impaired energetic capacity of aging skeletal muscle. This age-associated finding is consistent with previous studies in patients with peripheral vascular disease. For example, Kemp and colleagues showed oxygen usage outpaces delivery in these patients during and after exercise, based on combined measurements of ^31^P MRS and NIRS (Kemp et al., 2001, 2002). Our results suggest that, similar to the previous study, reduced oxygen delivery, as inferred from resting muscle perfusion, could in part explain reduced oxidative capacity in healthy aging. Indeed, this observation is important both for fundamental understanding of the physiology of muscle aging and for identifying potential targets for treatment of frailty and loss of muscle strength with aging.

Another interesting finding of this work is the significant correlation of resting blood volume with aerobic capacity (peak VO_2_). This result suggests that lower muscle perfusion in older adults contributes to the aerobic capacity decline with aging, at least in part, likely because of a reduction in the number of capillaries at rest and increase in microvascular resistance, which reduces muscle oxygen consumption and eventually leads to whole-body aerobic capacity decline with age. These findings are consistent with a recent publication by Prior and colleagues (Prior et al., 2016) that showed a significant correlation between VO_2_ max and muscle capillarization (capillary-to-fiber ratio) in a cohort of sarcopenic middle-age and old adults (age = 61 ± 1 years). Our results demonstrate that, even in an extremely healthy population in the absence of diseases or frailty, resting muscle perfusion contributes to the decline in peak VO_2_ and this contribution is mediated by mitochondrial function as shown in Figure 4. This illustration suggests a hypothesis that the decline of resting muscle perfusion limits the delivery of adequate oxygen and substrate to the mitochondria network and creates a deficiency in mitochondrial function. As a result of this deficit muscle oxygen consumption decreases within a contraction, which ultimately leads to a decline in whole-body aerobic capacity. However, because of the complexity and interdependence of age-associated decline in both peripheral capillary density and mitochondrial function that may occur simultaneously with age, a definite conclusion cannot be stated. Further investigation on this issue should be performed, specifically looking at these associations in a longitudinal perspective and also examining dynamic changes in perfusion during exercise.

The major strength of this study is the careful screening of participants to have a healthy cohort with no central or peripheral cardiovascular disease, or frailty as mentioned above. Another strength is the non-invasive technique that we used for muscle perfusion quantification *in vivo* at the level of the microvasculature system that estimates an index of the blood volume in muscle tissue, and is independent of the muscle oxygenation and metabolic capacity of the skeletal muscle. However, given the complexity of this method, more efforts to describe this methodology and possibly validate it against other approaches are required.

Furthermore, a longitudinal study is essential to verify the mediator role of muscle oxidative capacity between resting muscle perfusion and whole-body aerobic capacity. To overcome this limitation, we intend to repeat MRI based measures of perfusion in the GESTALT cohort biannually over the next 10 years. Furthermore, one of the main features of aging is that most physiological deficits are best assessed during challenge. For example, a number of studies found that endothelial reactivity is dampened in older individuals in response to exercise and reperfusion after ischemia (Hearon and Dinenno, 2016). Thus, we predict that microvascular function also plays a major role, separately or jointly with muscle oxidative capacity, in the decline of muscle perfusion at rest; therefore we intend to measure the changes of muscle perfusion following knee extension exercise inside of the MRI apparatus in subsequent visits of the GESTALT study. In spite of these caveats, the current study remains important since it is the first empirical demonstration that age-associated impairment of resting muscle perfusion in a healthy adult population, as a basal capacity of microvascular function, strongly predicts both muscle oxidative capacity and whole-body aerobic capacity.

## Conclusion

In summary, this study shows a decline of the *in vivo* oxidative capacity of skeletal muscle with age in an extremely healthy population, which indicates that this decline is not due to a disease process but rather to aging. Additionally, the significant correlation between resting muscle perfusion and whole-body aerobic capacity mediated by muscle oxidative capacity, independent of age, suggests new interventions that improve muscle perfusion might attenuate mitochondrial dysfunction with aging as well as the age-associated decline of aerobic capacity and frailty in sarcopenic and older adults.

## Ethics Statement

This study was carried out in accordance with the recommendations of “the IRB of the National Institute of Environmental Health Science (Research Triangle Park, North Carolina)” with written informed consent from all subjects. All subjects gave written informed consent in accordance with the Declaration of Helsinki. The protocol was approved by the “IRB of the National Institute of Environmental Health Science.”

## Author Contributions

FA, DC, CB, KF, RS, DR, and LF were involved in the study conception, and design. FA, DR, and LF performed the collection of and/or analysis of data. FA and LF wrote the manuscript. All the authors approved the final version of the manuscript.

## Conflict of Interest Statement

The authors declare that the research was conducted in the absence of any commercial or financial relationships that could be construed as a potential conflict of interest.
