# Supplementary material for: The Role of Muscle Perfusion in the Age-Associated Decline of Mitochondrial Function in Healthy Individuals
Source: Front Physiol. 2019 Apr 12;10:427. doi: 10.3389/fphys.2019.00427 (PMC6473080; doi:10.3389/fphys.2019.00427)
Supplement: Supplementary file 2 [file Table_1.DOCX]

Supplementary Material

# Data

Dataset of the current study including the characteristics of the sample, was separately uploaded in Excel file as supplementary material.

# Supplementary Figures

##
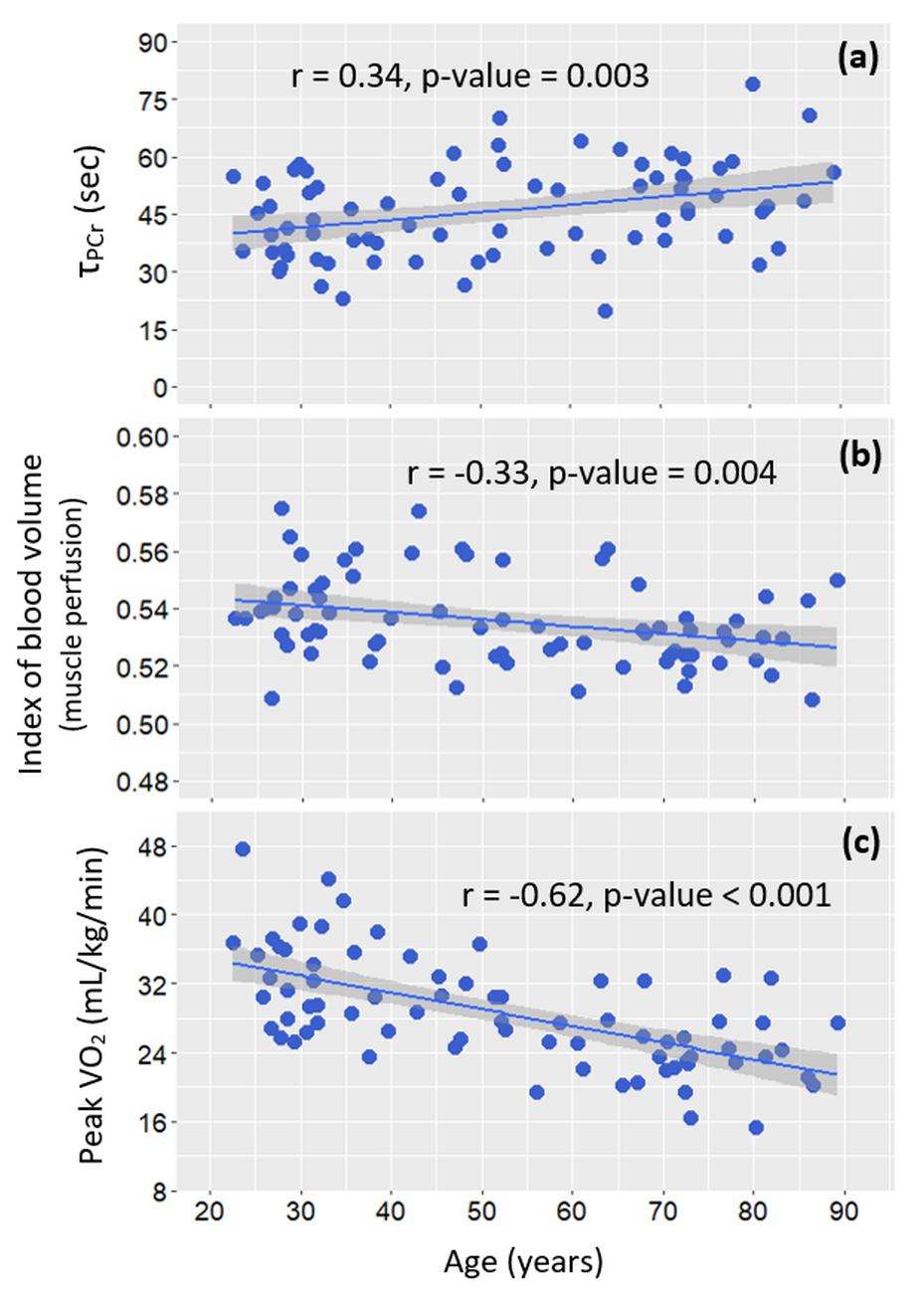


**Supplementary Figure 1.** Scatter plot of relationships between age and (a) post-exercise recovery time of phosphocreatine (τ_PCr_), (b) index of microvascular blood volume which reflects resting muscle perfusion, (c) whole-body aerobic capacity (Peak VO_2_). Linear regression line and summary statistic (Pearson’s correlation and p-value) are shown.
